# 1,2,3,3′,4′,6′-Hexaacetyl-4,6-*O*-benzyl­idenesucrose

**DOI:** 10.1107/S1600536811002546

**Published:** 2011-01-26

**Authors:** Marco A. Brito-Arias, Miguel Soto-Ortega, Efrén V. García-Báez

**Affiliations:** a Unidad Profesional Interdisciplinaria de Biotecnología, Instituto Politécnico Nacional, Avenida Acueducto s/n, Barrio La Laguna Ticomán, México DF 07340, Mexico

## Abstract

In the title compound, C_31_H_38_O_17_, the 1,3-dioxane and pyran­oside rings both show ^4^ *C*_1_ chair conformations while for the d-fructofuran­oside moiety an envelop 3*E* conformation is observed. The phenyl ring is oriented almost perpendicular to the 1,3-dioxane ring [dihedral angle = 79.3 (2)°], and the acetate groups are equatorial for the pyran­oside ring and axial for the furan­oside ring. The analysis of potential hydrogen bonds shows both intra- and inter­molecular C—H⋯O contacts to be present.

## Related literature

For sucrose and sucralose, see: Robyt (1998 ▶); Fairclough *et al.* (1995 ▶). For sucrose derivatives as potential pharmaceutically active substances, see: El Sayed & El Nemr (2005 ▶); Furneaux *et al.* (1993 ▶). For details of *O*-glycosidic bonds, see: Brito-Arias *et al.* (2007 ▶). For conformational analysis of five and six-membered rings, see: Cremer & Pople (1975 ▶); Evans & Boeyens (1989 ▶).
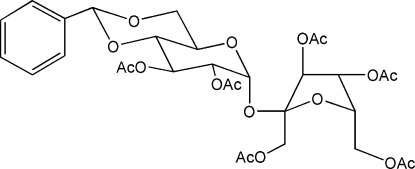


## Experimental

### 

#### Crystal data

C_31_H_38_O_17_
*M* *_r_* = 682.61
Orthorhombic, 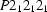

*a* = 8.2018 (2) Å
*b* = 18.6416 (3) Å
*c* = 22.0994 (5) Å
*V* = 3378.88 (12) Å^3^
*Z* = 4
Mo *K*α radiation
μ = 0.11 mm^−1^
*T* = 293 K
0.40 × 0.30 × 0.20 mm

#### Data collection

Oxford Diffraction CrysAlis CCD diffractometer
10881 measured reflections
5582 independent reflections
4757 reflections with *I* > 2σ(*I*)
*R*_int_ = 0.019

#### Refinement

*R*[*F* ^2^ > 2σ(*F* ^2^)] = 0.036
*wR*(*F* ^2^) = 0.091
*S* = 1.03
5582 reflections
440 parameters
H-atom parameters constrained
Δρ_max_ = 0.25 e Å^−3^
Δρ_min_ = −0.15 e Å^−3^

### 

Data collection: *CrysAlis CCD* (Oxford Diffraction, 2003 ▶); cell refinement: *CrysAlis CCD*; data reduction: *CrysAlis RED* (Oxford Diffraction, 2003 ▶); program(s) used to solve structure: *SHELXS97* (Sheldrick, 2008 ▶); program(s) used to refine structure: *SHELXL97* (Sheldrick, 2008 ▶); molecular graphics: *SHELXTL* (Sheldrick, 2008 ▶) and *Mercury* (Macrae *et al.*, 2006 ▶); software used to prepare material for publication: *SHELXL97*, *PLATON* (Spek, 2009 ▶) and *WinGX2003* (Farrugia, 1999 ▶).

## Supplementary Material

Crystal structure: contains datablocks I, global. DOI: 10.1107/S1600536811002546/su2240sup1.cif

Structure factors: contains datablocks I. DOI: 10.1107/S1600536811002546/su2240Isup2.hkl

Additional supplementary materials:  crystallographic information; 3D view; checkCIF report

## Figures and Tables

**Table 1:** Hydrogen-bond geometry (Å, °)

*D*—H⋯*A*	*D*—H	H⋯*A*	*D*⋯*A*	*D*—H⋯*A*
C1—H1⋯O10	0.98	2.49	3.108 (3)	121
C1—H1⋯O15^i^	0.98	2.56	3.399 (3)	143
C15—H15⋯O17	0.98	2.49	3.309 (3)	141
C25—H25*A*⋯O12^ii^	0.96	2.51	3.343 (3)	145
C29—H29*C*⋯O12^iii^	0.96	2.56	3.412 (4)	148
C31—H31*C*⋯O14^iv^	0.96	2.56	3.504 (4)	168

Symmetry codes: (i) 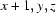; (ii) 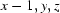; (iii) 
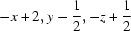
; (iv) 
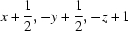
.

**Fig. 1.**
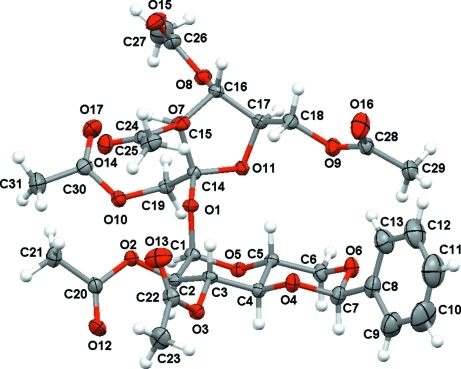
The molecular structure of the title comound, showing the atom-labelling scheme. Displacement ellipsoids are drawn at the 30% probability level.

**Fig. 2.**
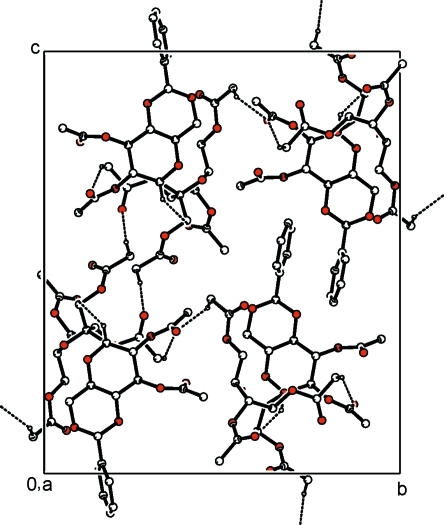
A view of the crystal packing along the a-axis of the title compound. The C-H···O interactions are shown as dotted lines [H-atoms not involved in C-H···O interactions have been omitted for clarity].

**Fig. 3.**
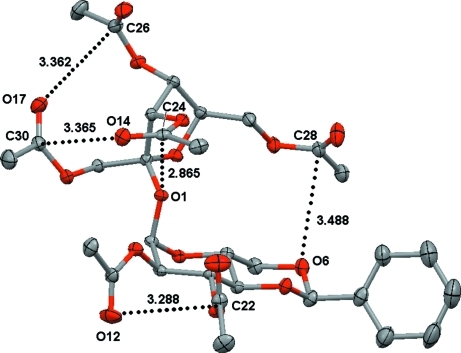
A view of the intramolecular C=O···C(acetyl) contacts in the title compound [H-atoms have been omitted for clarity].

## supplementary crystallographic information

### Comment

Sucrose is an abundant and low cost sugar mainly used as natural edulcorant (Robyt, 1998), and when substituted by chlorine at certain positions as the artificial edulcorant sucralose (Fairclough *et al.*, 1995). Despite this important usefulness sucrose has not been exploited sufficiently as synton for preparing modified derivatives containing isosteric substituents which could eventually lead us to pharmaceutical active substances (El Sayed & El Nemr, 2005; Furneaux *et al.* 1993). As a part of a strategy directed toward the preparation of modified sucrose derivatives we have prepared the title compound, a protected sucrose derivative which contains a benzylidene group at the 4 and 6 position of the glucopyranoside moiety and is fully acetylated at the remaining hydroxyl positions. This intermediate will allow us to functionalize the hydroxyl position at the pyranoside ring after deprotection of the benzylidene protecting group under mild conditions.

The title compound (Fig.1), shows two ^4^C_1_ chair conformations belonging to the pyranoside and the 1,3-dioxane rings with puckering parameters (Cremer & Pople, 1975) Q = 0.572 (2) Å, θ = 7.7 (2)°, φ = 304.4 (15)° and Q = 0.577 (2) Å, θ = 0.0 (2)°, φ = 46 (7)°; both values being in agreement with a chair conformation. Also the angular disposition for the endocyclic bond C1—O5—C5 of 111.83 (15)° is in agreement with ^4^C_1_ conformations having the substituents positioned at equatorial positions. The phenyl group is oriented almost perpendicular to the 1,3-dioxane and the acetate groups attached to the pyranoside ring are in equatorial positions. The α-anomeric C1—O1 bond value of 1.412 (2)Å is more enlongated than the reference value of 1.385 (4)Å for O-glycosidic bonds (Brito-Arias *et al.*, 2007). For the furanoside ring the torsion angle values are 2.2 (2)° for C15—C14—O11—C17 revealing these elements to be almost in the plane and -28.9 (2)° for C14—C15—C16—C17 indicating an envelop *exo* E for C16, in agreement with a syn-periplanar conformation (Evans & Boeyens, 1989).

The analysis of potential hydrogen bonds shows different intramolecular and intermolecular C—H···O contacts are present (Table 1). The molecular packing is shown in Fig. 2, and some of the intramolecular O···C(acetyl) contacts, in the range 3.488 to 2.865 Å, are indicated in Fig. 3.

### Experimental

The title compound was prepared by following a two step sequence starting from D-sucrose, which was treated with benzaldehyde dimethylacetal in dimethylformamide, followed by peracetylation under acetic anhydride-pyridine conditions. After purification by column chromatography the title compound was obtained as a white crystalline solid. ^1^H NMR data are available in the archived CIF.

### Refinement

The absolute configuration of the structure could not determined by the X-ray analysis [Flack  parameter = -0.3 (8)], but was already known from the configuration of the starting material, D-sucrose. H-atoms were placed in calculated positions and treated as riding atoms: C—H = 0.93, 0.98, 0.97 and 0.96 Å for CH(aromatic), CH(methine), CH_2_ and CH_3_, respectively, with U_iso_(H) = k × U_eq_(C), where k = 1.5 for CH_3_ H-atoms and k = 1.2 for all other H-atoms.

### Crystal data

**Table:**

C_31_H_38_O_17_	*F*(000) = 1440
*M**_r_* = 682.61	*D*_x_ = 1.342 Mg m^−^^3^
Orthorhombic, *P*2_1_2_1_2_1_	Mo *K*α radiation, λ = 0.71073 Å
Hall symbol: P 2ac 2ab	Cell parameters from 600 reflections
*a* = 8.2018 (2) Å	θ = 20–25°
*b* = 18.6416 (3) Å	µ = 0.11 mm^−^^1^
*c* = 22.0994 (5) Å	*T* = 293 K
*V* = 3378.88 (12) Å^3^	Block, colourless
*Z* = 4	0.40 × 0.30 × 0.20 mm

### Data collection

**Table:**

Oxford Diffraction CrysAlis CCD diffractometer	4757 reflections with *I* > 2σ(*I*)
Radiation source: fine-focus sealed tube	*R*_int_ = 0.019
graphite	θ_max_ = 25.0°, θ_min_ = 2.4°
φ and ω scans	*h* = −9→9
10881 measured reflections	*k* = −22→18
5582 independent reflections	*l* = −22→26

### Refinement

**Table:**

Refinement on *F*^2^	Hydrogen site location: inferred from neighbouring sites
Least-squares matrix: full	H-atom parameters constrained
*R*[*F*^2^ > 2σ(*F*^2^)] = 0.036	*w* = 1/[σ^2^(*F*_o_^2^) + (0.0523*P*)^2^ + 0.4056*P*] where *P* = (*F*_o_^2^ + 2*F*_c_^2^)/3
*wR*(*F*^2^) = 0.091	(Δ/σ)_max_ = 0.002
*S* = 1.03	Δρ_max_ = 0.25 e Å^−^^3^
5582 reflections	Δρ_min_ = −0.15 e Å^−^^3^
440 parameters	Extinction correction: *SHELXL97* (Sheldrick, 2008), Fc^*^=kFc[1+0.001xFc^2^λ^3^/sin(2θ)]^-1/4^
0 restraints	Extinction coefficient: 0.0023 (5)
Primary atom site location: structure-invariant direct methods	Absolute structure: Flack, H. D. (1983). *Acta Cryst.* A39, 876–881.
Secondary atom site location: difference Fourier map	Flack parameter: −0.3 (8)

### Special details

**Table:**

Experimental. Spectroscopic ^1^H NMR analysis in CDCl_3_, 300 MHz, δ, p.p.m. shows: 2.0–2.1(6 s, 18H acetates), 3.6–3.8 (m, 2*H*-16,17), 4.1–4.4 (m, 7*H*-5,6,18,19), 4.8 (dd, 1*H*-2), 5.3(t, 1*H*-4), 5.4(d, 1*H*-15), 5.5(s, 1*H*-benzyl), 5.6(t, 1*H*-3), 5.7(d, 1*H*-1), 7.3–7.4(m, 5H aromatics).
Geometry. Bond distances, angles *etc*. have been calculated using the rounded fractional coordinates. All su's are estimated from the variances of the (full) variance-covariance matrix. The cell e.s.d.'s are taken into account in the estimation of distances, angles and torsion angles
Refinement. Refinement of *F*^2^ against ALL reflections. The weighted *R*-factor *wR* and goodness of fit *S* are based on *F*^2^, conventional *R*-factors *R* are based on *F*, with *F* set to zero for negative *F*^2^. The threshold expression of *F*^2^ > σ(*F*^2^) is used only for calculating *R*-factors(gt) *etc*. and is not relevant to the choice of reflections for refinement. *R*-factors based on *F*^2^ are statistically about twice as large as those based on *F*, and *R*- factors based on ALL data will be even larger.

### Fractional atomic coordinates and isotropic or equivalent isotropic displacement parameters (Å^2^)

**Table:**

	*x*	*y*	*z*	*U*_iso_*/*U*_eq_	
O1	0.73111 (16)	0.17519 (7)	0.30944 (6)	0.0333 (4)	
O2	0.86704 (18)	0.30014 (7)	0.32503 (7)	0.0406 (5)	
O3	1.00795 (18)	0.32682 (7)	0.20241 (7)	0.0401 (5)	
O4	0.9344 (2)	0.20942 (7)	0.12304 (7)	0.0443 (5)	
O5	0.96831 (16)	0.12242 (7)	0.27017 (6)	0.0353 (5)	
O6	0.9303 (2)	0.08528 (8)	0.10929 (7)	0.0504 (5)	
O7	0.42743 (17)	0.19824 (7)	0.30318 (6)	0.0382 (5)	
O8	0.36078 (19)	0.02962 (8)	0.38025 (7)	0.0444 (5)	
O9	0.5580 (2)	0.01360 (9)	0.18192 (7)	0.0515 (6)	
O10	0.74965 (19)	0.16117 (8)	0.43694 (7)	0.0441 (5)	
O11	0.64870 (17)	0.05572 (7)	0.30428 (7)	0.0377 (4)	
O12	1.0842 (3)	0.37072 (12)	0.33670 (11)	0.0865 (9)	
O13	0.7900 (3)	0.39817 (10)	0.21369 (12)	0.0833 (9)	
O14	0.4859 (2)	0.28084 (8)	0.37415 (8)	0.0552 (6)	
O15	0.1459 (2)	0.09224 (12)	0.41209 (9)	0.0705 (7)	
O16	0.4388 (3)	0.06265 (12)	0.10214 (9)	0.0840 (8)	
O17	0.5304 (3)	0.13599 (12)	0.49345 (9)	0.0731 (8)	
C1	0.9033 (2)	0.17528 (10)	0.30896 (10)	0.0344 (6)	
C2	0.9547 (2)	0.25001 (10)	0.28831 (9)	0.0342 (6)	
C3	0.9194 (3)	0.26387 (10)	0.22161 (10)	0.0340 (6)	
C4	0.9826 (3)	0.20166 (10)	0.18466 (9)	0.0361 (6)	
C5	0.9142 (3)	0.13166 (10)	0.20908 (9)	0.0347 (6)	
C6	0.9739 (3)	0.07081 (11)	0.17039 (11)	0.0458 (8)	
C7	0.9946 (3)	0.15103 (12)	0.08835 (11)	0.0508 (8)	
C8	0.9397 (4)	0.16212 (14)	0.02379 (12)	0.0613 (10)	
C9	1.0530 (5)	0.1828 (2)	−0.01927 (16)	0.0947 (16)	
C10	1.0007 (8)	0.1938 (3)	−0.07948 (19)	0.130 (3)	
C11	0.8399 (9)	0.1862 (3)	−0.0937 (2)	0.129 (2)	
C12	0.7291 (7)	0.1660 (2)	−0.05162 (19)	0.1057 (19)	
C13	0.7789 (5)	0.15374 (19)	0.00733 (14)	0.0813 (14)	
C14	0.6485 (3)	0.11941 (10)	0.33997 (9)	0.0314 (6)	
C15	0.4675 (2)	0.14265 (10)	0.34567 (9)	0.0334 (6)	
C16	0.3740 (3)	0.07573 (11)	0.32814 (10)	0.0379 (7)	
C17	0.4873 (3)	0.03726 (12)	0.28463 (10)	0.0391 (7)	
C18	0.4578 (3)	0.05827 (14)	0.21980 (11)	0.0509 (8)	
C19	0.7226 (3)	0.09899 (11)	0.40039 (10)	0.0366 (7)	
C20	0.9452 (3)	0.35802 (12)	0.34689 (11)	0.0468 (8)	
C21	0.8321 (4)	0.40377 (13)	0.38178 (12)	0.0580 (9)	
C22	0.9294 (3)	0.39056 (11)	0.20049 (11)	0.0487 (8)	
C23	1.0434 (4)	0.44780 (12)	0.18165 (15)	0.0703 (10)	
C24	0.4529 (3)	0.26677 (11)	0.32286 (11)	0.0402 (7)	
C25	0.4346 (3)	0.31905 (13)	0.27285 (12)	0.0559 (8)	
C26	0.2401 (3)	0.04389 (14)	0.41925 (11)	0.0509 (8)	
C27	0.2438 (5)	−0.00724 (19)	0.47071 (14)	0.0857 (14)	
C28	0.5332 (3)	0.02021 (14)	0.12248 (11)	0.0491 (8)	
C29	0.6319 (4)	−0.03097 (15)	0.08675 (12)	0.0603 (9)	
C30	0.6439 (3)	0.17443 (15)	0.48229 (12)	0.0510 (8)	
C31	0.6844 (4)	0.24131 (19)	0.51508 (14)	0.0777 (12)	
H1	0.94339	0.16705	0.35016	0.0413*	
H2	1.07174	0.25597	0.29557	0.0410*	
H3	0.80215	0.27032	0.21503	0.0408*	
H4	1.10184	0.20050	0.18705	0.0434*	
H5	0.79477	0.13321	0.20806	0.0416*	
H6A	1.09139	0.06637	0.17394	0.0549*	
H6B	0.92478	0.02612	0.18353	0.0549*	
H7	1.11397	0.14992	0.09022	0.0610*	
H9	1.16182	0.18930	−0.00874	0.1136*	
H10	1.07559	0.20612	−0.10930	0.1562*	
H11	0.80557	0.19516	−0.13305	0.1542*	
H12	0.62016	0.16029	−0.06227	0.1268*	
H13	0.70281	0.13969	0.03619	0.0977*	
H15	0.44170	0.15735	0.38716	0.0400*	
H16	0.26775	0.08664	0.31002	0.0454*	
H17	0.47181	−0.01463	0.28886	0.0469*	
H18A	0.34372	0.05174	0.20961	0.0611*	
H18B	0.48561	0.10834	0.21379	0.0611*	
H19A	0.65003	0.06634	0.42141	0.0439*	
H19B	0.82535	0.07445	0.39372	0.0439*	
H21A	0.89344	0.43589	0.40696	0.0869*	
H21B	0.76373	0.37413	0.40665	0.0869*	
H21C	0.76555	0.43100	0.35440	0.0869*	
H23A	1.07607	0.43993	0.14047	0.1054*	
H23B	1.13776	0.44709	0.20733	0.1054*	
H23C	0.99033	0.49354	0.18490	0.1054*	
H25A	0.32536	0.33750	0.27267	0.0838*	
H25B	0.45654	0.29572	0.23499	0.0838*	
H25C	0.51018	0.35782	0.27848	0.0838*	
H27A	0.35180	−0.00885	0.48739	0.1286*	
H27B	0.21358	−0.05416	0.45674	0.1286*	
H27C	0.16841	0.00820	0.50128	0.1286*	
H29A	0.65424	−0.01087	0.04762	0.0904*	
H29B	0.57280	−0.07507	0.08206	0.0904*	
H29C	0.73279	−0.04019	0.10737	0.0904*	
H31A	0.58619	0.26275	0.53026	0.1167*	
H31B	0.73752	0.27412	0.48800	0.1167*	
H31C	0.75590	0.23046	0.54824	0.1167*	

### Atomic displacement parameters (Å^2^)

**Table:**

	*U*^11^	*U*^22^	*U*^33^	*U*^12^	*U*^13^	*U*^23^
O1	0.0262 (7)	0.0325 (7)	0.0412 (8)	−0.0004 (6)	0.0000 (6)	0.0034 (6)
O2	0.0395 (8)	0.0333 (7)	0.0489 (9)	−0.0014 (7)	0.0031 (7)	−0.0078 (7)
O3	0.0446 (8)	0.0261 (7)	0.0497 (9)	−0.0036 (6)	0.0056 (7)	0.0012 (6)
O4	0.0603 (9)	0.0355 (7)	0.0370 (9)	0.0018 (8)	0.0028 (8)	−0.0014 (7)
O5	0.0338 (8)	0.0310 (7)	0.0412 (9)	0.0052 (6)	−0.0009 (7)	0.0037 (6)
O6	0.0671 (10)	0.0372 (8)	0.0469 (10)	0.0008 (8)	−0.0025 (9)	−0.0078 (7)
O7	0.0347 (7)	0.0380 (8)	0.0419 (9)	0.0044 (7)	−0.0049 (7)	0.0020 (7)
O8	0.0434 (8)	0.0408 (8)	0.0490 (10)	−0.0032 (7)	0.0079 (8)	0.0029 (7)
O9	0.0530 (10)	0.0620 (10)	0.0395 (10)	0.0060 (9)	−0.0050 (9)	−0.0087 (8)
O10	0.0430 (9)	0.0517 (9)	0.0376 (9)	−0.0053 (8)	−0.0030 (7)	−0.0051 (7)
O11	0.0332 (7)	0.0336 (7)	0.0462 (9)	0.0005 (6)	−0.0023 (7)	−0.0068 (7)
O12	0.0566 (12)	0.0907 (15)	0.1121 (18)	−0.0310 (12)	0.0178 (12)	−0.0535 (14)
O13	0.0627 (13)	0.0481 (10)	0.139 (2)	0.0146 (10)	0.0125 (14)	0.0245 (12)
O14	0.0667 (11)	0.0432 (9)	0.0556 (11)	0.0024 (8)	0.0027 (10)	−0.0081 (8)
O15	0.0507 (10)	0.0970 (15)	0.0637 (13)	0.0153 (12)	0.0120 (10)	−0.0033 (11)
O16	0.1039 (16)	0.0990 (15)	0.0491 (11)	0.0343 (15)	−0.0173 (12)	−0.0057 (11)
O17	0.0702 (13)	0.0932 (14)	0.0558 (12)	−0.0139 (13)	0.0200 (10)	−0.0090 (10)
C1	0.0293 (10)	0.0339 (10)	0.0401 (12)	0.0002 (9)	−0.0027 (9)	0.0029 (9)
C2	0.0263 (10)	0.0335 (10)	0.0427 (12)	−0.0002 (9)	0.0012 (10)	−0.0043 (9)
C3	0.0313 (10)	0.0271 (9)	0.0436 (12)	−0.0032 (9)	0.0021 (10)	0.0032 (9)
C4	0.0341 (10)	0.0348 (10)	0.0395 (12)	−0.0015 (9)	0.0018 (10)	−0.0013 (9)
C5	0.0331 (10)	0.0309 (10)	0.0400 (13)	0.0025 (9)	0.0004 (10)	0.0004 (9)
C6	0.0532 (14)	0.0334 (11)	0.0507 (14)	0.0066 (10)	−0.0044 (12)	−0.0049 (10)
C7	0.0613 (15)	0.0443 (13)	0.0469 (15)	0.0029 (12)	0.0069 (13)	−0.0062 (11)
C8	0.087 (2)	0.0494 (14)	0.0475 (15)	0.0023 (15)	0.0097 (17)	−0.0070 (12)
C9	0.113 (3)	0.107 (3)	0.064 (2)	−0.006 (3)	0.017 (2)	−0.006 (2)
C10	0.172 (5)	0.166 (5)	0.053 (3)	−0.009 (4)	0.024 (3)	0.016 (3)
C11	0.182 (5)	0.147 (4)	0.057 (3)	0.005 (4)	−0.011 (3)	0.000 (3)
C12	0.133 (4)	0.113 (3)	0.071 (3)	0.008 (3)	−0.031 (3)	−0.009 (2)
C13	0.099 (3)	0.090 (2)	0.055 (2)	−0.004 (2)	−0.0155 (18)	−0.0001 (17)
C14	0.0335 (10)	0.0283 (9)	0.0324 (11)	−0.0015 (9)	0.0014 (10)	0.0007 (9)
C15	0.0317 (10)	0.0347 (10)	0.0337 (11)	0.0000 (9)	−0.0017 (9)	0.0038 (9)
C16	0.0309 (10)	0.0426 (11)	0.0401 (13)	−0.0047 (9)	−0.0023 (10)	0.0011 (10)
C17	0.0351 (11)	0.0399 (11)	0.0422 (13)	−0.0064 (10)	−0.0005 (10)	−0.0037 (10)
C18	0.0417 (13)	0.0657 (15)	0.0454 (14)	0.0054 (12)	−0.0051 (12)	−0.0104 (12)
C19	0.0345 (11)	0.0369 (11)	0.0385 (12)	0.0026 (10)	−0.0001 (10)	0.0046 (9)
C20	0.0546 (15)	0.0436 (12)	0.0423 (13)	−0.0086 (12)	0.0026 (12)	−0.0088 (10)
C21	0.0783 (18)	0.0436 (13)	0.0520 (16)	0.0016 (13)	0.0075 (15)	−0.0094 (12)
C22	0.0619 (16)	0.0325 (12)	0.0516 (15)	0.0000 (11)	−0.0031 (14)	0.0048 (10)
C23	0.090 (2)	0.0320 (12)	0.089 (2)	−0.0106 (14)	0.0151 (19)	0.0066 (13)
C24	0.0321 (11)	0.0362 (11)	0.0523 (15)	0.0044 (10)	0.0084 (12)	−0.0007 (11)
C25	0.0529 (14)	0.0431 (12)	0.0717 (17)	0.0081 (12)	0.0089 (14)	0.0133 (12)
C26	0.0443 (14)	0.0585 (15)	0.0499 (15)	−0.0088 (14)	0.0085 (13)	−0.0049 (12)
C27	0.104 (3)	0.092 (2)	0.061 (2)	−0.011 (2)	0.0239 (19)	0.0173 (17)
C28	0.0531 (14)	0.0553 (14)	0.0390 (14)	−0.0040 (13)	−0.0067 (13)	−0.0040 (11)
C29	0.0673 (17)	0.0643 (16)	0.0492 (15)	−0.0045 (15)	−0.0032 (13)	−0.0125 (13)
C30	0.0457 (14)	0.0653 (16)	0.0419 (14)	0.0026 (14)	−0.0047 (12)	−0.0006 (13)
C31	0.080 (2)	0.088 (2)	0.065 (2)	−0.0055 (18)	−0.0028 (18)	−0.0296 (18)

### Geometric parameters (Å, °)

**Table:**

O1—C1	1.412 (2)	C20—C21	1.477 (4)
O1—C14	1.413 (2)	C22—C23	1.479 (4)
O2—C2	1.431 (2)	C24—C25	1.481 (3)
O2—C20	1.345 (3)	C26—C27	1.484 (4)
O3—C3	1.444 (2)	C28—C29	1.480 (4)
O3—C22	1.352 (3)	C30—C31	1.480 (4)
O4—C4	1.425 (3)	C1—H1	0.9800
O4—C7	1.420 (3)	C2—H2	0.9800
O5—C1	1.411 (2)	C3—H3	0.9800
O5—C5	1.432 (2)	C4—H4	0.9800
O6—C6	1.423 (3)	C5—H5	0.9800
O6—C7	1.412 (3)	C6—H6A	0.9700
O7—C15	1.437 (2)	C6—H6B	0.9700
O7—C24	1.366 (2)	C7—H7	0.9800
O8—C16	1.441 (3)	C9—H9	0.9300
O8—C26	1.339 (3)	C10—H10	0.9300
O9—C18	1.439 (3)	C11—H11	0.9300
O9—C28	1.335 (3)	C12—H12	0.9300
O10—C19	1.430 (3)	C13—H13	0.9300
O10—C30	1.348 (3)	C15—H15	0.9800
O11—C14	1.425 (2)	C16—H16	0.9800
O11—C17	1.435 (3)	C17—H17	0.9800
O12—C20	1.186 (3)	C18—H18A	0.9700
O13—C22	1.189 (3)	C18—H18B	0.9700
O14—C24	1.195 (3)	C19—H19A	0.9700
O15—C26	1.198 (3)	C19—H19B	0.9700
O16—C28	1.195 (3)	C21—H21A	0.9600
O17—C30	1.200 (4)	C21—H21B	0.9600
C1—C2	1.525 (3)	C21—H21C	0.9600
C2—C3	1.524 (3)	C23—H23A	0.9600
C3—C4	1.510 (3)	C23—H23B	0.9600
C4—C5	1.520 (3)	C23—H23C	0.9600
C5—C6	1.503 (3)	C25—H25A	0.9600
C7—C8	1.510 (4)	C25—H25B	0.9600
C8—C9	1.385 (5)	C25—H25C	0.9600
C8—C13	1.377 (5)	C27—H27A	0.9600
C9—C10	1.413 (6)	C27—H27B	0.9600
C10—C11	1.363 (10)	C27—H27C	0.9600
C11—C12	1.354 (8)	C29—H29A	0.9600
C12—C13	1.384 (5)	C29—H29B	0.9600
C14—C15	1.552 (3)	C29—H29C	0.9600
C14—C19	1.516 (3)	C31—H31A	0.9600
C15—C16	1.515 (3)	C31—H31B	0.9600
C16—C17	1.517 (3)	C31—H31C	0.9600
C17—C18	1.505 (3)		
			
O1···O2	2.6052 (19)	C22···O2	3.268 (3)
O1···O7	2.5313 (19)	C22···O12	3.288 (3)
O1···O10	2.834 (2)	C23···O11^iii^	3.244 (3)
O1···O14	3.157 (2)	C24···O1	2.865 (3)
O1···C24	2.865 (3)	C25···O12^iv^	3.343 (3)
O2···O3	2.988 (2)	C26···O17	3.362 (3)
O2···O13	3.129 (3)	C29···O6	3.307 (3)
O2···O1	2.6052 (19)	C29···O12^i^	3.412 (4)
O2···C22	3.268 (3)	C30···O14	3.365 (3)
O3···O4	2.869 (2)	C30···C15	3.400 (3)
O3···O12	3.141 (3)	C1···H19B	2.7300
O3···O2	2.988 (2)	C2···H5	3.1000
O3···C20	3.286 (3)	C12···H9^ix^	3.0600
O4···O3	2.869 (2)	C12···H23A^ix^	3.0500
O5···O11	2.9977 (19)	C13···H9^ix^	3.0800
O6···C29	3.307 (3)	C14···H18B	3.1000
O7···C18	3.204 (3)	C15···H18B	2.9900
O7···O11	3.2176 (19)	C16···H19A	3.0700
O7···O1	2.5313 (19)	C19···H1	2.4700
O8···O11	2.938 (2)	C23···H19B^iii^	3.0800
O8···C19	3.268 (3)	C26···H15	2.7800
O9···O11	2.912 (2)	C28···H21C^xi^	3.0100
O10···C1	3.108 (3)	C30···H15	2.7000
O10···O1	2.834 (2)	H1···O10	2.4900
O11···O8	2.938 (2)	H1···O15^ii^	2.5600
O11···C23^i^	3.244 (3)	H1···C19	2.4700
O11···C5	3.343 (3)	H1···H19B	2.2000
O11···O7	3.2176 (19)	H2···O12	2.3300
O11···O5	2.9977 (19)	H3···O1	2.8000
O11···O9	2.912 (2)	H3···O13	2.3900
O12···C25^ii^	3.343 (3)	H3···H5	2.5600
O12···C22	3.288 (3)	H4···H6A	2.5200
O12···O3	3.141 (3)	H4···H7	2.3400
O12···C29^iii^	3.412 (4)	H5···O1	2.4300
O13···O2	3.129 (3)	H5···O11	2.8400
O13···C20	3.293 (4)	H5···C2	3.1000
O14···C30	3.365 (3)	H5···H3	2.5600
O14···O1	3.157 (2)	H5···H18B	2.5800
O14···C14	3.377 (3)	H6A···H4	2.5200
O15···C15	3.162 (2)	H6A···H7	2.4300
O15···C1^iv^	3.399 (3)	H6A···H18A^ii^	2.2300
O17···C26	3.362 (3)	H7···H4	2.3400
O17···C21^v^	3.286 (4)	H7···H6A	2.4300
O17···C15	3.309 (3)	H7···H9	2.3400
O1···H5	2.4300	H9···H7	2.3400
O1···H3	2.8000	H9···C12^vi^	3.0600
O3···H11^vi^	2.9100	H9···C13^vi^	3.0800
O5···H16^ii^	2.6900	H11···O3^ix^	2.9100
O5···H23C^i^	2.6200	H11···H25B^vi^	2.5800
O6···H13	2.6700	H12···H23A^ix^	2.5700
O6···H29C	2.8500	H13···O6	2.6700
O7···H18B	2.6300	H15···O10	2.7600
O8···H19A	2.6300	H15···O14	2.3500
O10···H15	2.7600	H15···O15	2.7700
O10···H1	2.4900	H15···O17	2.4900
O11···H23B^i^	2.6900	H15···C26	2.7800
O11···H5	2.8400	H15···C30	2.7000
O12···H25A^ii^	2.5100	H15···H19A	2.5200
O12···H2	2.3300	H16···O5^iv^	2.6900
O12···H29C^iii^	2.5600	H16···O15	2.4700
O13···H25C	2.8100	H16···H18A	2.3900
O13···H17^vii^	2.6900	H17···O13^xi^	2.6900
O13···H3	2.3900	H18A···O16	2.5100
O14···H29B^vii^	2.9000	H18A···H6A^iv^	2.2300
O14···H15	2.3500	H18A···H16	2.3900
O14···H31C^v^	2.5600	H18B···O7	2.6300
O15···H16	2.4700	H18B···O16	2.6400
O15···H19B^iv^	2.6800	H18B···C14	3.1000
O15···H15	2.7700	H18B···C15	2.9900
O15···H1^iv^	2.5600	H18B···H5	2.5800
O16···H27C^viii^	2.7400	H19A···O8	2.6300
O16···H18B	2.6400	H19A···O17	2.2800
O16···H18A	2.5100	H19A···C16	3.0700
O17···H15	2.4900	H19A···H15	2.5200
O17···H19A	2.2800	H19B···O15^ii^	2.6800
O17···H21A^v^	2.8100	H19B···C1	2.7300
C1···O10	3.108 (3)	H19B···H1	2.2000
C1···O15^ii^	3.399 (3)	H19B···C23^i^	3.0800
C5···O11	3.343 (3)	H21A···O17^x^	2.8100
C9···C12^vi^	3.533 (6)	H21B···H31B	2.6000
C9···C13^vi^	3.576 (5)	H21C···C28^vii^	3.0100
C12···C9^ix^	3.533 (6)	H23A···C12^vi^	3.0500
C13···C9^ix^	3.576 (5)	H23A···H12^vi^	2.5700
C14···O14	3.377 (3)	H23B···O11^iii^	2.6900
C15···C30	3.400 (3)	H23C···O5^iii^	2.6200
C15···O17	3.309 (3)	H25A···O12^iv^	2.5100
C15···O15	3.162 (2)	H25B···H11^ix^	2.5800
C18···O7	3.204 (3)	H25C···O13	2.8100
C19···O8	3.268 (3)	H27C···O16^xii^	2.7400
C20···C22	3.294 (3)	H29B···O14^xi^	2.9000
C20···O13	3.293 (4)	H29C···O6	2.8500
C20···O3	3.286 (3)	H29C···O12^i^	2.5600
C21···O17^x^	3.286 (4)	H31B···H21B	2.6000
C22···C20	3.294 (3)	H31C···O14^x^	2.5600
			
C1—O1—C14	118.95 (15)	C4—C3—H3	111.00
C2—O2—C20	119.21 (16)	O4—C4—H4	109.00
C3—O3—C22	118.93 (17)	C3—C4—H4	109.00
C4—O4—C7	109.97 (16)	C5—C4—H4	109.00
C1—O5—C5	111.83 (15)	O5—C5—H5	110.00
C6—O6—C7	112.44 (17)	C4—C5—H5	110.00
C15—O7—C24	115.57 (16)	C6—C5—H5	110.00
C16—O8—C26	116.84 (18)	O6—C6—H6A	110.00
C18—O9—C28	115.60 (19)	O6—C6—H6B	110.00
C19—O10—C30	117.95 (18)	C5—C6—H6A	110.00
C14—O11—C17	111.48 (16)	C5—C6—H6B	110.00
O1—C1—O5	112.44 (15)	H6A—C6—H6B	108.00
O1—C1—C2	106.24 (14)	O4—C7—H7	110.00
O5—C1—C2	110.59 (16)	O6—C7—H7	110.00
O2—C2—C1	106.73 (15)	C8—C7—H7	110.00
O2—C2—C3	110.01 (15)	C8—C9—H9	121.00
C1—C2—C3	113.06 (16)	C10—C9—H9	121.00
O3—C3—C2	109.05 (17)	C9—C10—H10	120.00
O3—C3—C4	107.01 (18)	C11—C10—H10	120.00
C2—C3—C4	109.12 (16)	C10—C11—H11	119.00
O4—C4—C3	110.09 (17)	C12—C11—H11	119.00
O4—C4—C5	108.91 (16)	C11—C12—H12	120.00
C3—C4—C5	109.92 (18)	C13—C12—H12	120.00
O5—C5—C4	108.90 (16)	C8—C13—H13	120.00
O5—C5—C6	110.17 (17)	C12—C13—H13	120.00
C4—C5—C6	109.02 (18)	O7—C15—H15	111.00
O6—C6—C5	108.36 (17)	C14—C15—H15	111.00
O4—C7—O6	111.01 (18)	C16—C15—H15	111.00
O4—C7—C8	107.54 (19)	O8—C16—H16	113.00
O6—C7—C8	108.5 (2)	C15—C16—H16	113.00
C7—C8—C9	119.2 (3)	C17—C16—H16	113.00
C7—C8—C13	121.3 (3)	O11—C17—H17	109.00
C9—C8—C13	119.5 (3)	C16—C17—H17	109.00
C8—C9—C10	118.9 (4)	C18—C17—H17	109.00
C9—C10—C11	119.7 (5)	O9—C18—H18A	110.00
C10—C11—C12	121.3 (5)	O9—C18—H18B	110.00
C11—C12—C13	119.6 (5)	C17—C18—H18A	110.00
C8—C13—C12	120.8 (4)	C17—C18—H18B	110.00
O1—C14—O11	110.38 (16)	H18A—C18—H18B	108.00
O1—C14—C15	106.99 (15)	O10—C19—H19A	109.00
O1—C14—C19	114.41 (18)	O10—C19—H19B	109.00
O11—C14—C15	106.18 (17)	C14—C19—H19A	109.00
O11—C14—C19	106.13 (16)	C14—C19—H19B	109.00
C15—C14—C19	112.47 (18)	H19A—C19—H19B	108.00
O7—C15—C14	111.55 (15)	C20—C21—H21A	109.00
O7—C15—C16	108.13 (15)	C20—C21—H21B	109.00
C14—C15—C16	103.51 (16)	C20—C21—H21C	109.00
O8—C16—C15	108.96 (17)	H21A—C21—H21B	109.00
O8—C16—C17	105.70 (17)	H21A—C21—H21C	109.00
C15—C16—C17	103.96 (18)	H21B—C21—H21C	109.00
O11—C17—C16	105.06 (17)	C22—C23—H23A	109.00
O11—C17—C18	111.96 (19)	C22—C23—H23B	109.00
C16—C17—C18	112.5 (2)	C22—C23—H23C	109.00
O9—C18—C17	108.15 (19)	H23A—C23—H23B	109.00
O10—C19—C14	110.87 (16)	H23A—C23—H23C	109.00
O2—C20—O12	123.4 (2)	H23B—C23—H23C	109.00
O2—C20—C21	110.6 (2)	C24—C25—H25A	109.00
O12—C20—C21	126.0 (2)	C24—C25—H25B	109.00
O3—C22—O13	123.8 (2)	C24—C25—H25C	109.00
O3—C22—C23	110.0 (2)	H25A—C25—H25B	109.00
O13—C22—C23	126.3 (2)	H25A—C25—H25C	109.00
O7—C24—O14	122.8 (2)	H25B—C25—H25C	109.00
O7—C24—C25	111.3 (2)	C26—C27—H27A	109.00
O14—C24—C25	125.9 (2)	C26—C27—H27B	109.00
O8—C26—O15	122.8 (2)	C26—C27—H27C	109.00
O8—C26—C27	110.5 (2)	H27A—C27—H27B	109.00
O15—C26—C27	126.7 (3)	H27A—C27—H27C	109.00
O9—C28—O16	122.0 (2)	H27B—C27—H27C	109.00
O9—C28—C29	112.5 (2)	C28—C29—H29A	109.00
O16—C28—C29	125.5 (2)	C28—C29—H29B	109.00
O10—C30—O17	122.8 (2)	C28—C29—H29C	109.00
O10—C30—C31	112.0 (2)	H29A—C29—H29B	109.00
O17—C30—C31	125.2 (3)	H29A—C29—H29C	109.00
O1—C1—H1	109.00	H29B—C29—H29C	109.00
O5—C1—H1	109.00	C30—C31—H31A	109.00
C2—C1—H1	109.00	C30—C31—H31B	109.00
O2—C2—H2	109.00	C30—C31—H31C	109.00
C1—C2—H2	109.00	H31A—C31—H31B	109.00
C3—C2—H2	109.00	H31A—C31—H31C	109.00
O3—C3—H3	111.00	H31B—C31—H31C	109.00
C2—C3—H3	111.00		
			
C14—O1—C1—O5	73.7 (2)	O5—C1—C2—C3	51.6 (2)
C14—O1—C1—C2	−165.23 (16)	O2—C2—C3—O3	75.5 (2)
C1—O1—C14—O11	−77.8 (2)	O2—C2—C3—C4	−167.97 (17)
C1—O1—C14—C15	167.07 (16)	C1—C2—C3—O3	−165.34 (15)
C1—O1—C14—C19	41.8 (2)	C1—C2—C3—C4	−48.8 (2)
C20—O2—C2—C1	137.53 (18)	O3—C3—C4—O4	−68.8 (2)
C20—O2—C2—C3	−99.5 (2)	O3—C3—C4—C5	171.19 (17)
C2—O2—C20—O12	1.1 (3)	C2—C3—C4—O4	173.30 (17)
C2—O2—C20—C21	178.19 (18)	C2—C3—C4—C5	53.3 (2)
C22—O3—C3—C2	−97.8 (2)	O4—C4—C5—O5	177.60 (16)
C22—O3—C3—C4	144.32 (19)	O4—C4—C5—C6	57.4 (2)
C3—O3—C22—O13	−0.1 (4)	C3—C4—C5—O5	−61.7 (2)
C3—O3—C22—C23	178.2 (2)	C3—C4—C5—C6	178.05 (19)
C7—O4—C4—C3	179.74 (18)	O5—C5—C6—O6	−174.82 (17)
C7—O4—C4—C5	−59.7 (2)	C4—C5—C6—O6	−55.4 (2)
C4—O4—C7—O6	61.4 (2)	O4—C7—C8—C9	107.1 (3)
C4—O4—C7—C8	179.9 (2)	O4—C7—C8—C13	−71.4 (3)
C5—O5—C1—O1	58.8 (2)	O6—C7—C8—C9	−132.8 (3)
C5—O5—C1—C2	−59.80 (19)	O6—C7—C8—C13	48.8 (3)
C1—O5—C5—C4	65.5 (2)	C7—C8—C9—C10	−179.4 (3)
C1—O5—C5—C6	−175.03 (16)	C13—C8—C9—C10	−0.9 (5)
C7—O6—C6—C5	57.8 (2)	C7—C8—C13—C12	178.2 (3)
C6—O6—C7—O4	−61.1 (2)	C9—C8—C13—C12	−0.3 (5)
C6—O6—C7—C8	−179.1 (2)	C8—C9—C10—C11	2.1 (7)
C24—O7—C15—C14	−88.8 (2)	C9—C10—C11—C12	−2.1 (8)
C24—O7—C15—C16	158.04 (18)	C10—C11—C12—C13	1.0 (7)
C15—O7—C24—O14	−9.4 (3)	C11—C12—C13—C8	0.3 (6)
C15—O7—C24—C25	170.49 (17)	O1—C14—C15—O7	19.1 (2)
C26—O8—C16—C15	83.2 (2)	O1—C14—C15—C16	135.10 (16)
C26—O8—C16—C17	−165.66 (19)	O11—C14—C15—O7	−98.83 (17)
C16—O8—C26—O15	0.2 (3)	O11—C14—C15—C16	17.2 (2)
C16—O8—C26—C27	−179.2 (2)	C19—C14—C15—O7	145.51 (16)
C28—O9—C18—C17	173.1 (2)	C19—C14—C15—C16	−98.46 (19)
C18—O9—C28—O16	3.0 (4)	O1—C14—C19—O10	49.9 (2)
C18—O9—C28—C29	−175.9 (2)	O11—C14—C19—O10	171.83 (17)
C30—O10—C19—C14	102.9 (2)	C15—C14—C19—O10	−72.5 (2)
C19—O10—C30—O17	0.9 (4)	O7—C15—C16—O8	−158.16 (16)
C19—O10—C30—C31	−178.5 (2)	O7—C15—C16—C17	89.49 (18)
C17—O11—C14—O1	−113.43 (19)	C14—C15—C16—O8	83.41 (19)
C17—O11—C14—C15	2.2 (2)	C14—C15—C16—C17	−28.9 (2)
C17—O11—C14—C19	122.06 (19)	O8—C16—C17—O11	−83.85 (19)
C14—O11—C17—C16	−20.8 (2)	O8—C16—C17—C18	154.14 (19)
C14—O11—C17—C18	101.6 (2)	C15—C16—C17—O11	30.8 (2)
O1—C1—C2—O2	50.38 (19)	C15—C16—C17—C18	−91.2 (2)
O1—C1—C2—C3	−70.7 (2)	O11—C17—C18—O9	68.1 (2)
O5—C1—C2—O2	172.67 (14)	C16—C17—C18—O9	−173.87 (18)

Symmetry codes: (i) −*x*+2, *y*−1/2, −*z*+1/2; (ii) *x*+1, *y*, *z*; (iii) −*x*+2, *y*+1/2, −*z*+1/2; (iv) *x*−1, *y*, *z*; (v) *x*−1/2, −*y*+1/2, −*z*+1; (vi) *x*+1/2, −*y*+1/2, −*z*; (vii) −*x*+1, *y*+1/2, −*z*+1/2; (viii) −*x*+1/2, −*y*, *z*−1/2; (ix) *x*−1/2, −*y*+1/2, −*z*; (x) *x*+1/2, −*y*+1/2, −*z*+1; (xi) −*x*+1, *y*−1/2, −*z*+1/2; (xii) −*x*+1/2, −*y*, *z*+1/2.

### Hydrogen-bond geometry (Å, °)

**Table:**

*D*—H···*A*	*D*—H	H···*A*	*D*···*A*	*D*—H···*A*
C1—H1···O10	0.98	2.49	3.108 (3)	121
C1—H1···O15^ii^	0.98	2.56	3.399 (3)	143
C2—H2···O12	0.98	2.33	2.708 (3)	102
C3—H3···O13	0.98	2.39	2.725 (3)	100
C5—H5···O1	0.98	2.43	2.799 (3)	102
C15—H15···O17	0.98	2.49	3.309 (3)	141
C19—H19A···O17	0.97	2.28	2.682 (3)	104
C25—H25A···O12^iv^	0.96	2.51	3.343 (3)	145
C29—H29C···O12^i^	0.96	2.56	3.412 (4)	148
C31—H31C···O14^x^	0.96	2.56	3.504 (4)	168

Symmetry codes: (ii) *x*+1, *y*, *z*; (iv) *x*−1, *y*, *z*; (i) −*x*+2, *y*−1/2, −*z*+1/2; (x) *x*+1/2, −*y*+1/2, −*z*+1.
